# Biological Nitrification Inhibition (BNI): Phenotyping of a Core Germplasm Collection of the Tropical Forage Grass *Megathyrsus maximus* Under Greenhouse Conditions

**DOI:** 10.3389/fpls.2020.00820

**Published:** 2020-06-12

**Authors:** Daniel Villegas, Ashly Arevalo, Jonathan Nuñez, Johanna Mazabel, Guntur Subbarao, Idupulapati Rao, Jose De Vega, Jacobo Arango

**Affiliations:** ^1^International Center for Tropical Agriculture (CIAT), Cali, Colombia; ^2^Japan International Research Center for Agricultural Sciences (JIRCAS), Tsukuba, Japan; ^3^Earlham Institute, Norwich Research Park, Norwich, United Kingdom

**Keywords:** climate change, genetic diversity, livestock systems, plant-soil interactions, tropics

## Abstract

Modern intensively managed pastures that receive large external nitrogen (N) inputs account for high N losses in form of nitrate (NO_3_^–^) leaching and emissions of the potent greenhouse gas nitrous oxide (N_2_O). The natural plant capacity to shape the soil N cycle through exudation of organic compounds can be exploited to favor N retention without affecting productivity. In this study, we estimated the relationship between biological nitrification inhibition (BNI), N_2_O emissions and plant productivity for 119 germplasm accessions of Guineagrass (*Megathyrsus maximus*), an important tropical forage crop for livestock production. This relation was tested in a greenhouse experiment measuring BNI as (i) rates of soil nitrification; (ii) abundance of ammonia-oxidizing bacteria (AOB) and archaea (AOA); and (iii) the capacity of root tissue extracts to inhibit nitrification *in vitro*. We then measured N_2_O emissions, aboveground biomass and forage nutrition quality parameters. Reductions on nitrification activity ranging between 30 and 70% were found across the germplasm collection of *M. maximus*. Accessions with low nitrification rates showed a lower abundance of AOB as well as a reduction in N_2_O emissions compared to accessions of high nitrification rates. The BNI capacity was not correlated to N uptake of plants, suggesting that there may be intraspecific variation in the exploitation of different N sources in this grass species. A group of accessions (cluster) with the most desirable agronomic and environmental traits among the collection was identified for further field validation. These results provide evidence of the ability of *M. maximus* to suppress soil nitrification and N_2_O emissions and their relationship with productivity and forage quality, pointing a way to develop N conservative improved forage grasses for tropical livestock production.

## Introduction

Nitrogen (N) is the most limiting nutrient for plant growth in terrestrial ecosystems (Hopkins, 2000). Different mechanisms of niche separation have consequently surged during the evolution of plants to avoid competition and exploit different N sources and facilitate N accumulation (Norton and Ouyang, 2019).

Nitrification is the biological oxidation of ammonium (NH_4_^+^) to nitrite (NO_2_^–^), and subsequently to nitrate (NO_3_^–^) due to the action of a wide variety of microorganisms in the soil, such as bacteria, archaea, and fungi (Hayatsu et al., 2008). Although most plants have the capacity to utilize either NH_4_^+^ or NO_3_^–^, the latter is less stable in the soil due to repellency of negative charges of clays and soil organic matter. High levels of nitrification lead to leaching of NO_3_^–^ to groundwater, and under certain conditions of carbon (C) availability and oxygen (O_2_) limitation, it generates gaseous losses of the potent greenhouse gas nitrous oxide (N_2_O) via denitrification (Robertson and Groffman, 2015). In fact, agricultural ecosystems contribute approximately 40% of the N_2_O emissions generated in terrestrial environments (Davidson, 2009; Syakila and Kroeze, 2011; Prosser et al., 2019).

In natural ecosystems, the flux of N is regulated through the exploitation of several organic or mineral sources of this element (Smolander et al., 2012). In contrast, agricultural ecosystems are often subject to excessive N application and large N losses. Up to 70% of mineral N might be lost driven by rapid nitrification (Subbarao et al., 2012).

The NH_4_^+^/NH_3_ pool in the soil may be enriched by the application of mineral fertilizers, deposition of animal excreta, atmospheric NH_3_ deposition, biological N fixation, and mineralization of organic N sources. Particularly in grazing pastures, the N derived from animal excreta can represent inputs of 600–1200 kg of N ⋅ ha^–1^ (Hamonts et al., 2013) and additional N inputs due to fertilization vary from one farm to another in a range of zero to thousands of kilograms per year. Such huge amounts of N, far above the capacity for plant assimilation, are prone to be used by nitrifiers to produce polluting forms of N. Thus, the equilibrium of NH_4_^+^/NH_3_ production and consumption may significantly impact ecosystem sustainability.

Nitrification can be controlled through different approaches, limiting NH_4_^+^ availability (i.e., managing fertilization) or inhibiting nitrifiers with either synthetic or naturally released compounds (Norton and Ouyang, 2019).

Furthermore, plant-based strategies have been discovered that improve N cycling and reduce N losses. Abalos et al. (2018) suggested that plants with acquisitive resource-use strategies (i.e., successful in nutrient-rich environments) reduced emissions of N_2_O compared to conservative species and proposed that root length density and specific leaf area were key traits that regulate N_2_O emissions and biomass yield in managed grasslands. Similarly, Meier et al. (2017) observed that root exudates of the loblolly pine stimulated the microbial degradation of fast-cycling N pools increasing N availability.

Recently, the development of new methods to study N transformations in the soil has demonstrated that certain plants have the ability to significantly reduce nitrification through root exudation of biological inhibitors. Such compounds block the ammonia-monooxygenase and hydroxylamine-oxidoreductase enzymatic pathways in soil microorganisms, which are responsible for the two-step oxidation of NH_4_^+^ to NO_2_^–^, in which hydroxylamine acts as an intermediate form (Subbarao et al., 2006). This mechanism, named biological nitrification inhibition (BNI), has been proposed as a strategy to conserve N in nutrient-poor environments where specific hotspots of N exceed the uptake capacity of plants (Subbarao et al., 2013). This approach differs from abovementioned plant strategies since root exuded BNI compounds act directly inhibiting nitrifiers. Thus, net nitrification is diminished and NH_4_^+^-N is available in the soil for a longer time.

Sward composition has been shown to impact on the level of N losses, due to either specific plant functional traits or through rhizosphere effects. However, it has been proposed that there may be an energetic cost of root exudation of BNI compounds to compete for N that could affect agricultural yield (Coskun et al., 2017). Thus, the relation between BNI and biomass productivity is still not well understood.

Since the first discovery of BNI, a systematic screening for this ability started in different field crops such as rice, wheat, sorghum, and tropical and temperate forage crops (Subbarao et al., 2007, 2013; Tesfamariam et al., 2014; O’Sullivan et al., 2016; Sun et al., 2016; Nuñez et al., 2018).

*Megathyrsus maximus* (Jacq.) B.K. Simon & S.W.L. Jacobs, also widely known as *Panicum maximum* or Guineagrass, is a pasture grass native from subtropical Africa and considered one of the best species to improve beef and milk production (Aganga and Tshwenyane, 2004; Moran, 2005; Jank et al., 2014). The best cultivars are persistent and produce high amounts of nutritious and palatable forage especially in highly fertile soils (Souza, 1999). The area planted with this species in South America after its introduction has dramatically increased since the 1970s, due to its high yield in crop rotation systems, particularly with rice in Brazil, and to its high animal stocking capacity (Landers, 2007; Jank et al., 2014). In fact, only two commercial cultivars of this grass (cvs. Tanzania and Mombaza) are responsible for 10% of the total forages seed market in Brazil (in a total pasture area of 100 Mha) (Euclides et al., 2010; Fernandes et al., 2014). The desirable agronomic traits of this species make it a suitable forage grass for the tropics to achieve sustainable intensification of livestock systems, as the expected means to increase food production (Intergovernmental Panel on Climate Change [IPCC], 2019).

The first assessment of different plants for their BNI capacity was performed in 18 species of pasture grasses, and cereal and legume crops. That study reported significant BNI only in the grasses *Urochloa humidicola* (*Uh*) (syn. *Brachiaria humidicola*) and *U. decumbens* (syn. *B. decumbens*), which reduced nitrification up to 90%, and suggested the absence of such trait in the other species, including *M. maximus* (syn. *P. maximum*) (Subbarao et al., 2007). In most of the plant species included in that study, only one variety per species was evaluated. However, in the last decade, other researchers have assessed several genotypes from crops previously described as lacking BNI capacity. They found significant BNI ability and substantial intraspecific variation for this trait in rice, wheat, and sorghum (Pariasca Tanaka et al., 2010; Subbarao et al., 2013; Arevalo, 2016; O’Sullivan et al., 2016; Sun et al., 2016).

The genebank of the Genetic Resources Program of the International Center for Tropical Agriculture (CIAT) holds a germplasm collection of more than five hundred accessions of *M. maximus* collected during the second half of the 20th century mainly from the East Africa region. Out of these five hundred, 119 germplasm accessions were identified in the 1980s as genotypes with superior agronomic performance. These accessions now constitute a core collection for selection, and hybridization (breeding). *Megathyrsus maximus* shows vast morphological differences between cultivars in terms of biomass production, forage quality, and response to N availability (Aganga and Tshwenyane, 2004). Therefore, we hypothesize that this species shows a heterogeneous distribution of the BNI trait within the collection.

The objective of the present study was to explore the BNI capacity of different germplasm accessions of Guineagrass (*M. maximus*) toward the identification of genetic materials with environmental added value suitable for sustainable intensification of forage-based livestock systems in the tropics. Hence, to achieve this objective, key activities included (i) measurement of the BNI potential of a germplasm collection of 119 accessions of *M. maximus*; (ii) determination of the effect that high BNI genotypes exert in the structure of the soil nitrifier community (bacteria and archaea); (iii) evaluation of the relationship between nitrification inhibition and forage quality parameters associated with the N content of the plants, and (iv) phenotypic characterization of agronomic and environmental traits of the germplasm collection.

## Materials and Methods

### Experimental Design

A greenhouse trial was established in 2016 growing 119 germplasm accessions of *M. maximus* and the high BNI *U. humidicola* CIAT 16888 (*Uh*) as a positive control. All the plant genetic resources for this study were obtained from the genebank of CIAT in Palmira, Colombia. The trial was laid out following a complete randomized design with three replications. In each experimental unit, three plants of the same germplasm accession were seeded in pots with 6 kg of soil and sand mixed with a proportion of 2:1, respectively. The soil type was a Vertisol (Typic Pellustert; Instituto Geográfico Agustín Codazzi [IGAC], 1980) with a silty clay loam texture (50% clay) from the experimental field of CIAT. Three pots with soil and no plants were used as negative controls. The plants grew for 12 months and its maintenance included irrigation every second day and N application every 120 days in liquid form of (NH_4_)_2_SO_4_ at a rate of 90 kg ⋅ ha^–1^. Four commercial cultivars of *M. maximus* were included in the study, namely, the cvs. Mombaza (CIAT 6962), Agrosavia Sabanera (CIAT 6799), Vencedor (CIAT 26900), and Massai (CIAT 16021).

### Nitrification Rates of Soil

The nitrification rate (NR) of the soils where the 363 experimental units grew were determined following the microcosm incubation method (Karwat et al., 2017). Topsoil samples (0–10 cm) were collected using a soil auger, air-dried for 48 h and passed through a 2 mm sieve. To each of these, five subsamples of 3 g of soil were placed in 15 mL amber flasks and amended with 27 mM (NH_4_)_2_SO_4_. Soil moisture was maintained at 60% of field capacity during incubation. Extraction of mineral N was performed at different time points including a basal extraction (before incubation), and later at 1, 2, 4, 8, and 20 days after incubation at 25°C. The extraction was carried out shaking the soils with 30 mL of 1 M KCl at 200 rpm for 30 min and filtering using a filter paper Whatman grade 2. Quantification of inorganic N forms in soil extracts was performed by colorimetric methods and measurement of absorbance, from alkalinization with sodium salicylate at 410 nm for NO_3_^–^ and formation of the indophenol complex at 667 nm for NH_4_^+^ using a Synergy HT spectrophotometer. Nitrification rates were calculated as the slope of a linear regression between concentrations of NO_3_^–^ and incubation time.

### Nutrition Quality and Forage Productivity

Aboveground plant biomass was cut 15 cm above from the soil level for standardization of the trial. After 35 days of regrowth, the plant shoot material was harvested and oven-dried at 60°C for 72 h. Plant samples were ground and passed through a 1 mm sieve, and dry weight was recorded. Nutritional quality parameters of acid and neutral detergent fiber (ADF and NDF, respectively), *in vitro* dry matter digestibility (IVDMD) and N concentration in shoot tissue were determined by Near-Infrared Spectroscopy (NIRS) using a Foss DS 2500 spectrometer (FOSS NIRSystems Inc., Silver Spring, MD, United States). Crude protein (CP) was estimated multiplying the total N concentration in shoot tissue by a conversion factor of 6.25 (AOAC, 2002). Nitrogen uptake was calculated multiplying the N concentration by the yield of aboveground biomass.

Downstream analyses were based only on the most contrasting germplasm accessions according to NR of soils. The measurement of the specific BNI capacity of roots was done for two contrasting sets of ten accessions, whereas the determination of variation in abundance of nitrifier microbes and the measurement of nitrous oxide emissions was done for two contrasting sets of three germplasm accessions (already assessed for BNI potential of root tissue extracts).

### Abundance of Ammonia-Oxidizing Microbes

Soils where three accessions with the lowest NR and three with the highest grew were subjected to a new soil incubation experiment for 20 days. Soil samples were taken before incubation, and then after each time point (4, 8, and 20 days). Such samples were then frozen at -20°C, lyophilized, and total DNA was extracted using the FastDNA SPIN kit for soil (MP Biomedicals). Manufacturer’s instructions were modified with the addition of guanidine thiocyanate 5.5 M for the removal of humic substances. The copy number of *amoA* gene of ammonia-oxidizing archaea (AOA) and ammonia-oxidizing bacteria (AOB) was determined through quantitative PCR using a RotorGene Q thermocycler (QIAGEN). The reaction volume was 20 μL, containing 0.5 M of each pair of primers, 10 μL of Brilliant Sybr Green qPCR Master Mix, and 10 ng of DNA template. The primers combination was amoA-1F (5′-GGGGTTTCTACTGGTGGT-3′), amoA-2R (5′-CCCCTCKGSAAAGCCTTCTTC-3′) for AOB (Rotthauwe et al., 1997) and amoA19F (5′-ATGGTCTGGCTWAGACG-3′), amoA643R (5′-TCCCACTTWGACCARGCGGCCATCCA-3′) for AOA (Leininger et al., 2006). The amplification conditions of PCR were as follows: (1) 95°C for 10 min; (2) 95°C for 60 s; (3) 55°C for 30 s; (4) 72°C for 45 s; (5) plate read; (6) repeat steps 2–4 40 times; (7) melting curve from 65 to 95°C measuring each 0.2°C, maintained for 1 s.

### Inhibition of Nitrifying Activity *in vitro*

#### Collection of BNI Compounds

After 12 months since establishment, a destructive sampling was performed to measure the BNI potential of root tissue extracts. The plants were harvested from the pots and total biomass was split into shoot and root tissue. The roots were oven-dried at 60°C for 72 h and total dry weight was recorded before grinding. For the extraction of BNI compounds 1.5 mL of methanol were added to 100 mg of roots, followed by vigorous agitation and filtration with a 0.22 μm syringe filter. This extract was vacuum-dried and resuspended in 25 μL of dimethylsulfoxide (DMSO), and used as input for the luminescence assay.

#### Luminescence Assay

Root extracts were used to determine the specific BNI capacity of target accessions through the bioassay method (Subbarao et al., 2006; Nuñez et al., 2018). This procedure was conducted using a recombinant strain of *Nitrosomonas europaea* transformed to emit bioluminescence in the presence of decyl-aldehyde (Iizumi et al., 1998). The luminescence observed is highly correlated to NO_2_^–^ production (*R*^2^ = 0.94), therefore the extent of inhibition was quantified after the application of root extracts to the bacteria in terms of relative light units using a luminometer Glomax 20/20 (Promega). These values were then compared to a control consisting of vacuum-dried methanol resuspended in DMSO applied to the same bacteria. The percentage values of inhibition were transformed into Allylthiourea units (ATU), dividing the result by a factor of 80, which is the inhibition percentage of 0.22 μM of this synthetic inhibitor (Subbarao et al., 2006). The total BNI potential of each accession was finally calculated multiplying its specific BNI capacity (ATU ⋅ g of dry root^–1^) by the total root biomass in each pot.

### Nitrous Oxide Emissions From Soil

A small-scale measurement of N_2_O was performed in contrasting NR accessions described above. Gas samples were analyzed at four time points: basal (before N application), and then at 1, 4, and 7 days after fertilization in liquid form of (NH_4_)_2_SO_4_ at a rate of 90 kg ⋅ ha^–1^. Gas flux was measured using a portable Fourier Transform Infrared Spectroscopy (FTIR) Gas Analyzer (Gasmet DX4040) following the static chamber method (Teutscherova et al., 2018). Each pot containing three plants was covered with a plastic chamber and sealed with a rubber band before each measurement. On every measurement day, the concentrations of N_2_O were monitored continuously every 20 s for 10 min. Cumulative flux was calculated for each pot by linear interpolation of the concentration of N_2_O during the sampling period of 7 days.

#### Data Analysis

Statistical analyses were performed using R v3.4.4. Significant differences between treatments were assessed through analysis of variance and the Tukey HSD test for multiple comparisons. Data that deviated from a normal distribution (Shapiro Wilk test) were analyzed by Kruskal Wallis tests and the multiple comparison Dunn’s test using the R package “PMCMRplus” v1.0.0. Figures were constructed with “ggplot2” v2.2.1 and a cluster analysis integrating the variables measured for all germplasm accessions was performed using the libraries “FactoMineR” v1.39, “pca3d” v0.10, “rgl” v0.98.1, “car” v3.0-0, and “magick” v1.6.

## Results

### Nitrification Rates of Soil

**FIGURE 1:**
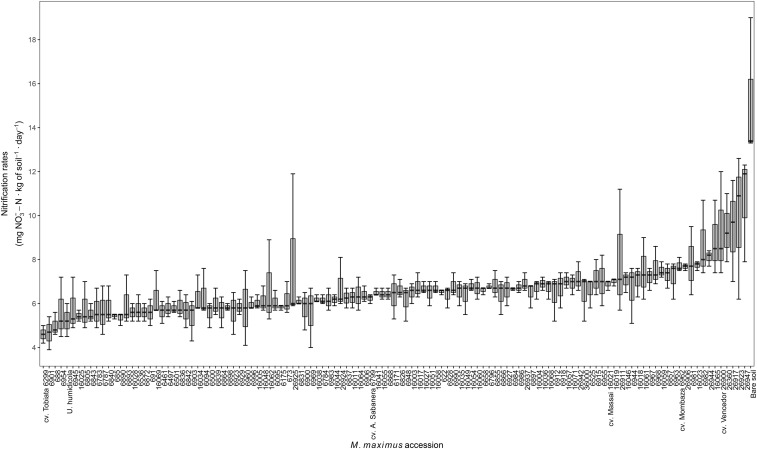
Nitrification rates of soil of 119 germplasm accessions of *M. maximus* after 12 months of planting in greenhouse conditions. Nitrification rates are expressed as the slope of a linear regression between concentration of NO_3_^–^ over time after 20 days of incubation (*n* = 3). *U. humidicola* and bare soil are positive and negative controls, respectively.

Moderate intraspecific variability was found among the germplasm collection of *M. maximus* for the ability to reduce nitrification in the soil [coefficient of variation (CV) = 20%]. Reductions observed were in the range of 30–70% (10.7–4.5 mg NO_3_^–^-N ⋅ kg of soil^–1^ ⋅ day^–1^) with respect to the bare soil control (15.2 mg NO_3_^–^-N ⋅ kg of soil^–1^ ⋅ day^–1^, considered as 100% for comparison purposes), whereas *Uh* showed reductions in NR of 55% (5.3 mg NO_3_^–^-N ⋅ kg of soil^–1^ ⋅ day^–1^). These values indicated that some accessions of *M. maximus* do exhibit higher capacity to inhibit soil NR compared to *Uh*, such as the accessions CIAT 688, CIAT 6901, and the cv. Tobiata, which in fact, showed the lowest NR among the entire collection. The commercial cultivars Mombaza, Sabanera, Vencedor and Massai, showed NR values of 7.7, 6.2, 9.3, and 6.9 mg NO_3_^–^-N ⋅ kg of soil^–1^ ⋅ day^–1^, respectively (Figure 1).

### Nitrification Rates and Ammonia-Oxidizing Microorganisms

**FIGURE 2:**
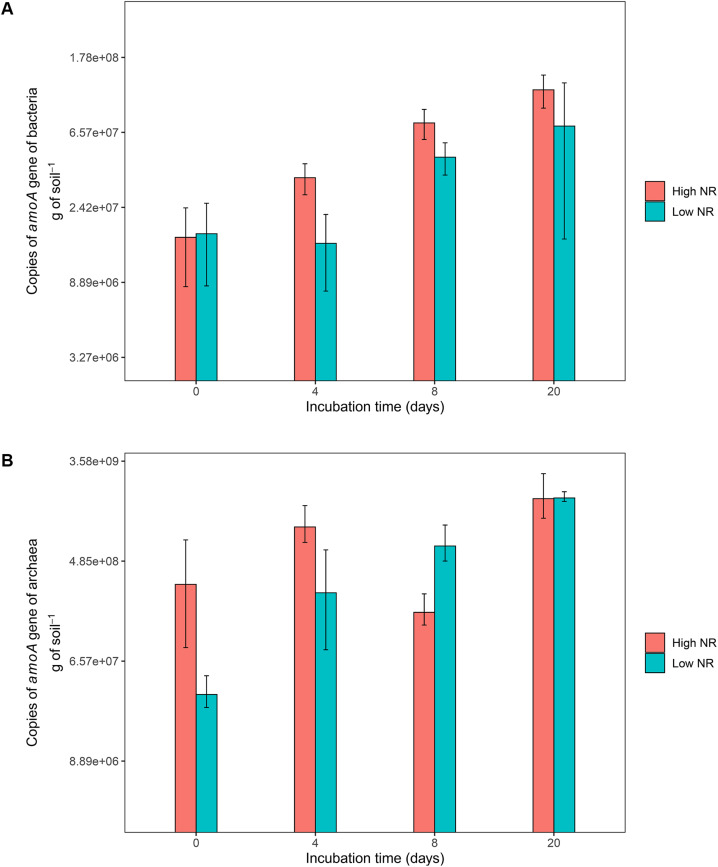
Abundance of ammonia-oxidizing bacteria **(A)** and archaea **(B)** over different incubation time points in soils. Blue color corresponds to accessions of low nitrification rates whereas red color corresponds to accessions of high nitrification rates.

The soils of low NR accessions of *M. maximus* (ten lowest according to Figure 1 and Supplementary Table 1) showed a lower abundance of AOB compared to soils where high NR accessions grew (ten highest in Figure 1 and Supplementary Table 1). Before incubation, soils collected from all accessions showed a similar abundance of AOB (close to 1.6 × 10^7^ copies of *amoA* gene ⋅ g of soil^–1^). During the following 20 days of incubation after N application (in the form of (NH_4_)_2_SO_4_), soils of high NR accessions showed on average 1.8 times more AOB abundance than soils from low NR accessions (*P* < 0.05) (Figure 2A). No statistical differences were found in the abundance of AOA for contrasting NR groups. Although soils of high NR accessions had almost ten times higher abundance of AOA than low NR accessions before incubation, no clear pattern of population growth was observed and both types of NR soils were equally abundant in AOA after 20 days of incubation (close to 7 × 10^8^ copies of *amoA* gene ⋅ g of soil^–1^) (Figure 2B).

### Nitrification Inhibition *in vitro*

The root extracts of the entire germplasm accessions evaluated showed significant potential to inhibit the activity of *N. europaea* under *in vitro* conditions. Low variability was found for specific BNI ranging from 112 to 143 ATU ⋅ g of dry root^–1^ (72–92% inhibition with respect to luminescence of bacteria without root tissue extracts, Supplementary Table 1). Thus, the differences in total BNI potential among germplasm accessions were only due to the variability of root biomass (2009–7097 ATU ⋅ g of dry root^–1^). No statistical differences were identified in total BNI potential of contrasting NR accessions (Supplementary Table 1).

### Emissions of Nitrous Oxide

**FIGURE 3:**
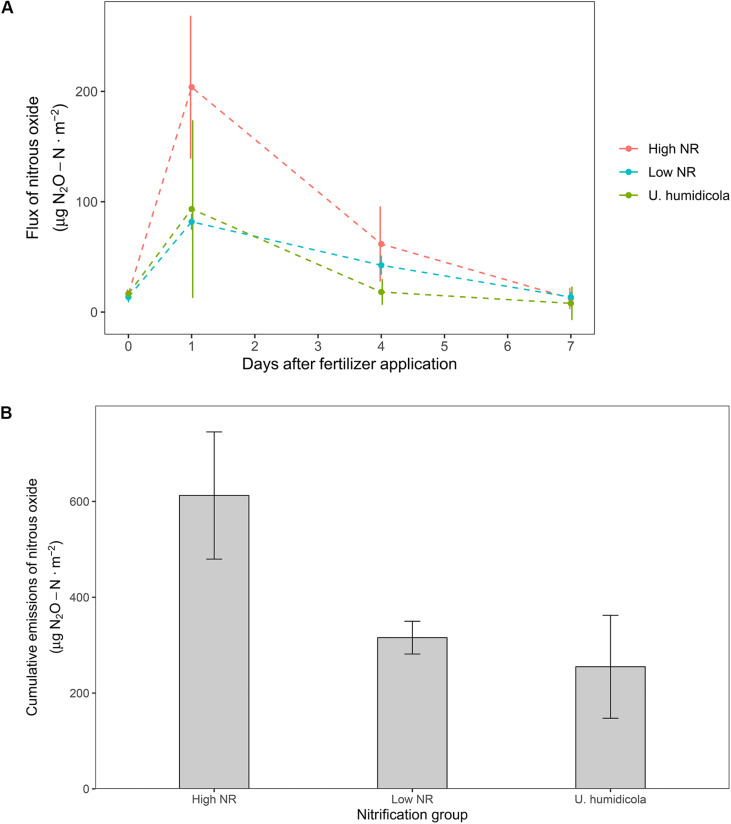
**(A)** Daily flux of N_2_O after fertilizer application in accessions of *M. maximus* of different nitrification rates and the high BNI control *U. humidicola*. **(B)** Cumulative emissions of N_2_O after the whole measurement period of seven days.

Soils from high NR accessions emitted more N_2_O than soils from low NR accessions. The peak of emissions during the sampling period was observed one day after N application, where high NR accessions emitted 2.5 times more N_2_O than low NR (Figure 3A). Taking into consideration the whole sampling period, the cumulative emissions of high NR accessions was 1.9 times higher than that of low NR (612 vs. 315 μg N_2_O-N⋅m^–2^) and 2.4 times higher than the high-BNI *Uh* control (255 μg N_2_O-N⋅m^–2^) (Figure 3B).

### Forage Quality and Nitrification Inhibition

The parameters of shoot biomass production, CP, and N uptake showed moderate variability among the germplasm collection. Coefficients of variation were 28, 17, and 23%, respectively. In contrast, these accessions of *M. maximus* did not differ significantly in the other nutritional quality parameters tested (CV = 3.0–6.6%). *In vitro* dry matter digestibility varied from 57.4 to 71.5%, whereas NDF and ADF showed values of 57.6–67.5% and 19.5–38.8%, respectively (Supplementary Table 2). Despite the ranges of these parameters which seem broad, the CV suggested that there is little variability of such traits within the population under greenhouse conditions.

No negative or positive effect was observed of NR on forage quality parameters directly related to N assimilation by the plant, namely shoot biomass production, CP, and N uptake [Coefficient of correlation (CC) = −0.08, 0.16, and 0.01, respectively].

**FIGURE 4:**
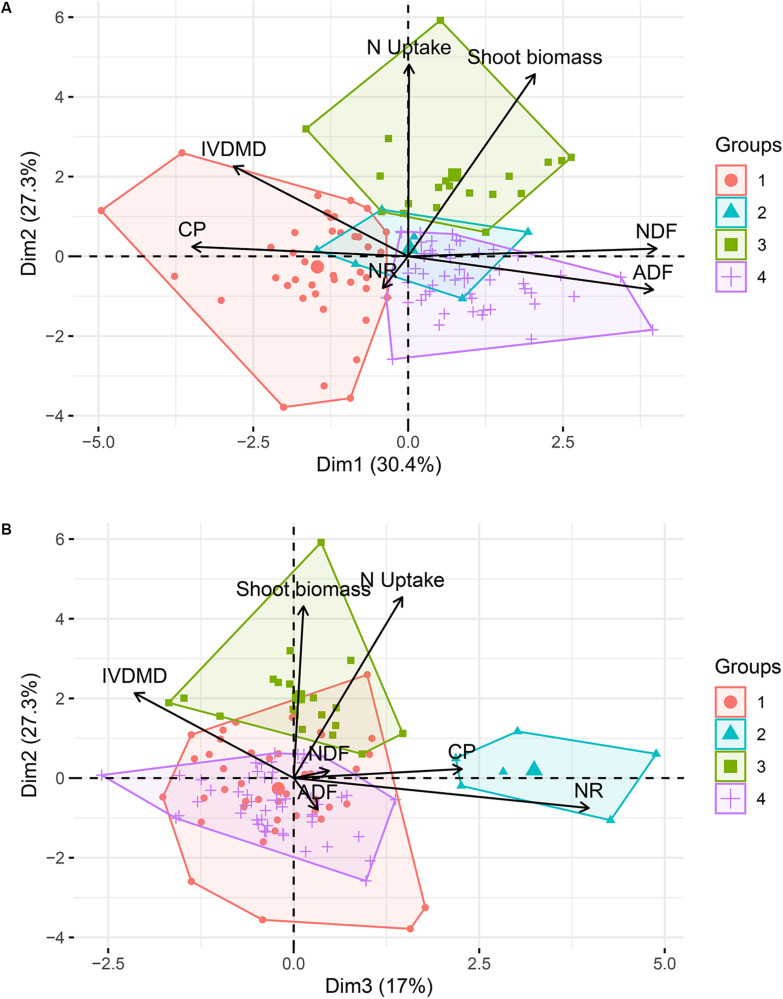
Cluster analysis based on principal components of the germplasm collection of *M. maximus*. Cumulative variance accounts for 74.7%. **(A)** View of the first two dimensions. **(B)** View of dimensions 2 and 3. NR, nitrification rates; CP, crude protein; IVDMD, *in vitro* dry matter digestibility; ADF, acid detergent fiber; NDF, neutral detergent fiber. Dimension 1 (30.4% of variation) mainly integrated the nutrition quality parameters of NDF, ADF, IVDMD, and CP. Dimension 2 (27.3% of variation) represented N Uptake and Shoot biomass. Dimension 3 (17% of variation) largely represented NR.

The cluster analysis, which accounted for 74.7% of the total variance, suggested a grouping of the evaluated germplasm collection of *M. maximus* into four subgroups [Cluster 1 (k1) = 41 accessions, k2 = 6, k3 = 19, and k4 = 52]. The first two dimensions discriminated the clusters 1, 3, and 4 (Figure 4A), whereas dimension 3, which was associated with NR discriminated the cluster 2 (Figure 4B). Differences between clusters were found for all the parameters evaluated. Cluster 3 (k3) grouped 19 germplasm accessions with the most desirable traits among the four clusters, with the highest biomass production and N uptake, and the lowest NR of the entire collection. On the other hand, cluster 4, which grouped 43% of the population, had the least desirable traits, showing the lowest content of CP and N uptake, but also the highest concentration of fibers (ADF and NDF). Cluster 1 showed the highest nutritional quality parameters, with the highest CP and IVDMD, but also the lowest content of fibers, and finally, cluster 2, discriminated by dimension 3, grouped six accessions with the highest NR values (Table 1).

**TABLE 1:** Characteristics of the four different clusters found within the collection of *M. maximus*.

Cluster	Number of accessions	Shoot biomass (g dry matter ⋅ pot^–1^)	NR (mg N-NO_3_ ⋅ kg soil^–1^ ⋅ day^–1^	CP (%)	N uptake (mg N ⋅ pot^–1^)	IVDMD (%)	NDF (%)	ADF (%)
1	42	15.3 ± 3.3a	6.3 ± 0.6a	**5.9 ± 0.6c**	143.5 ± 31.9a	**65.9 ± 1.6c**	**61.7 ± 1.5a**	**30.2 ± 1.5a**
2	6	19.1 ± 2b	9.5 ± 0.6b	5.8 ± 0.4bc	180.7 ± 26.9b	63.3 ± 2.6a	63.1 ± 1.9ab	31.5 ± 2ab
3	19	**23.5 ± 3.1c**	**6.1 ± 0.9a**	5.3 ± 0.6ab	**200.2 ± 30.3b**	65.5 ± 1.6bc	63.7 ± 1.2b	32.1 ± 1.2b
4	52	17 ± 1.9b	6.5 ± 0.6a	4.9 ± 0.5a	135.2 ± 16.7a	64.7 ± 1.5ab	63.8 ± 1.1b	32.8 ± 1.4b

**NR, nitrification rates, CP, crude protein, IVDMD, in vitro dry matter digestibility, ADF, acid detergent fiber, NDF, neutral detergent fiber. Different letters denote statistical differences according to analysis of variance and Tukey HSD test (α = 0.05). Bold numbers indicate the most desirable value for each trait within the clusters.**

## Discussion

### BNI Potential of *M. maximus*

This study provides the first evidence of capacity to reduce soil nitrification by tropical pasture grass accessions of *M. maximus* through the microcosm incubation method and bioassay of root tissue extracts. Previous research using only one genotype suggested the absence of detectable BNI activity (over 5 ATU) in this species (Subbarao et al., 2009; Espinosa et al., 2012). Nevertheless, in this study we were able to detect moderate variability among accessions tested, with potential to reduce nitrification up to 70% compared to bare soil and even exceeding the performance of the high BNI control *Uh* (which reduced NR 65% compared to bare soil control) in some genotypes where plant genetic background was the only source of variation within the population.

Comparable cases have been reported for cereal crops that were not expected to have BNI capacity based on evaluations of one or a few genotypes per species. O’Sullivan et al. (2016) evaluated root exudates and extracts from 96 wheat landraces, finding 26 lines with a statistically significant reduction in nitrification on two species of pure cultured bacteria. These results were then confirmed in a plant-soil experiment where after 4 weeks of growth, positive BNI landraces showed 25–45% reduction in NR compared to a bare soil. Similarly, Pariasca Tanaka et al. (2010) and Sun et al. (2016) evaluated 36 and 19 varieties of rice, respectively, both finding substantial variability in the capacity of root exudates to inhibit nitrification up to 50%.

Based on our results, *Megathyrsus maximus* cv. Tobiata can be considered an interesting model to study the BNI phenomenon due to its high capacity to reduce soil nitrification. According to Souza (1999), this is the most productive and vigorous cultivar of *M. maximus*. However, the adoption of this material for livestock production has been hindered by its susceptibility to spittlebug (Cercopidae: Hemiptera), difficult establishment in the field and complex management under grazing. Similarly, Dias Filho et al. (1992) stated that cv. Tobiata is susceptible to waterlogging and has low seed production compared to other cultivars of the species (Jank, 1995).

The cv. Tobiata showed similar productivity (24.6 g ⋅ pot^–1^ in Tobiata vs. 25.4 in Mombaza) under greenhouse conditions to cv. Mombaza (CIAT 6962), one of the grasses with the largest area planted in Brazil (Jank et al., 2008) for its simple management under grazing (Souza, 1999), and considered as one of the best genetic resources of *M. maximus*. This result is consistent with what was observed by Souza (1999) under field conditions, placing these forage yields among the highest values within the collection.

In terms of nutritional quality parameters, both cultivars showed a similar profile. Although cv. Tobiata had higher FDA than cv. Mombaza, the latter showed higher CP content, but also 58% higher NR. In this study, under greenhouse conditions, the cv. Tobiata was considered as the best performing cultivar, due to its low NR, desirable forage quality traits, which are similar, or superior to broadly planted genotypes of *M. maximus* in the tropics. Further breeding efforts should leverage on the desirable agronomic and environmental traits of the cv. Tobiata grass to improve tolerance to biotic and abiotic stresses.

### BNI Activity and Ammonia-Oxidizing Microbes in the Soil

The germplasm accessions with low NR showed a lower abundance of soil AOB. This result supports the theory proposed by Subbarao et al. (2015). Plants would release chemical compounds to the soil to suppress the activity of soil nitrifiers, possibly by blocking the ammonia-monooxygenase and the hydroxylamine-oxidoreductase enzymatic pathways, which are involved in the oxidation step of NH_4_^+^ into NO_2_^–^ (Subbarao et al., 2009). All evaluated accessions of *M. maximus* showed the capacity to reduce the oxidation of NH_4_^+^ over time. NR groups with contrasting phenotypes showed characteristic growth patterns of AOB reduction on time in a soil incubation experiment, whereas no difference was observed in the growth pattern of AOA across time within groups of accessions with contrasting NR.

Subbarao et al. (2009) reported an inhibitory effect of BNI compounds highly specific toward the AOB populations on field studies using the same soils from CIAT-Palmira. In their experiment, the abundance of AOB was consistent with the NH_4_^+^ oxidation rates observed in soil, whereas there was little effect on the distribution of the total bacterial population. However, all the accessions reduced the abundance of both, total and AOA in soil with respect to the control, independently of their BNI ability (Subbarao et al., 2009).

The dominance of AOA in the nitrification process in nutrient-poor acidic soils is well documented (He et al., 2012; Prosser and Nicol, 2012; Hu et al., 2014). However, plenty of factors such as ammonia availability, pH, organic matter, moisture content, and interaction of other environmental parameters have been proposed to drive ecological niche specialization among AOA and AOB in other ecosystems (Prosser and Nicol, 2008; Erguder et al., 2009; He et al., 2012; Zhalnina et al., 2012). In neutral to alkaline soils, changes in abundance and activity of both groups have been detected through molecular and sequencing-based methods (Nicol et al., 2008; Tourna et al., 2008). Our results are consistent with these published reports.

Bru et al. (2011) and Yao et al. (2013) evaluated a wide spectrum of temperate soils and found soil pH as the best predictor of the AOA-to-AOB ratio, consistently finding less abundance of AOB independently of the land use. These results are consistent with Leininger et al. (2006), who reported that AOA outnumber AOB, globally. In contrast, Prosser and Nicol (2008) found that in some agricultural soils, particularly in grasslands and pastures, the AOA-to-AOB ratios were significantly lower compared to natural ecosystems or even both groups could be equally abundant.

Shen et al. (2011) reported N levels and availability of NH_4_^+^ instead of soil pH as driving factors for AOA-to-AOB ratio in soil nitrifier communities. In their study in soils with similar content of organic matter, AOB outnumbered AOA in soils with pH of 5.4 at high fertilization levels, whereas the abundance of AOA exceeded AOB with either absent or low fertilization in soils of pH close to 7. According to Prosser et al. (2019), AOB are favored by high rates of supply, such as the addition of high concentrations of inorganic ammonium or urea, whereas AOA are successful in oligotrophic environments characterized by low pH and low ammonium supply (Robertson and Groffman, 2015).

In our study, despite AOA being more abundant than AOB, only the latter showed a characteristic growth pattern over time when incubated under favorable conditions for nitrification, as evidenced by the nitrate production response. In contrast, AOA growth seemed not to depend upon time or NH_4_^+^/NO_3_^–^ availability. This may support that AOB play a more pivotal role than AOA on nitrification in alkaline soils under high NH_4_^+^ availability. The observation that AOB growth correlated with soil NR suggests that BNI compounds released through *M. maximus* roots have a greater effect on AOB than AOA. These observations should be further confirmed through transcriptomic analysis of soil microbial communities for example.

### BNI Potential *in vitro* and Nitrification in the Soil

Subbarao et al. (2006) observed a linear correlation between BNI capacity measured through the luminescence bioassay and NR in *Uh* grasses (*R*^2^ = 0.97), whereas we did not find the same correlation for *M. maximus* grown in a similar soil. Likewise, Nuñez et al. (2018) evaluated the BNI potential of a biparental population (*n* = 117) of *Uh* finding no correlation between these two factors (*R*^2^ = 0.014).

Subbarao et al. (2013) suggested that although the quantification of the BNI ability through the bioassay is useful as an initial screening for large populations, these BNI compounds must be stable in the soil and persist over time to see an effective inhibitory response on nitrification in the soil. Therefore, it should not be expected that all BNI compounds released through the root system are equally effective in suppressing nitrification *in situ*. In fact, O’Sullivan et al. (2016) found no correlation between the level of BNI in the root tissue extracts of wheat and the one contained in root exudates, observing moderate to high BNI potential in root tissue independent of the inhibition caused by the exudates.

The main difference between the methodological approaches employed by Subbarao et al. (2006) and Nuñez et al. (2018) and this study was the input used for the bioassay. The initial study reported in 2006 used root exudates, whereas the other two performed the luminescence experiment with root tissue extracts. According to Nuñez et al. (2018) the relation between the BNI ability and NR can be affected by differences in the mechanisms of synthesis and release of BNI compounds between the genotypes evaluated. Moreover, independent of the total BNI potential found in certain genotypes, it is challenging to accurately estimate how much of the BNI compounds is exuded through the roots in an active form and how long they persist active in the soil system.

Although working with root exudates could be considered more straightforward to determine the true BNI capacity of these grasses as it might also account for the effect of dead root decomposition, the trap solutions used for plant exudation are sometimes highly artificial, not representing precisely the amount and quality of exudates in plant-soil systems (Coskun et al., 2017). Thus, in both cases the capacity of BNI compounds to reduce nitrification in soil could be over- or underestimated.

Lastly, within the limitations of extrapolating *in vitro* experiments to the field, it has been also proposed that the realization of bioassay exclusively with one strain of AOB (*N. europaea*) overlook the functional and taxonomic complexity of agricultural soils (Coskun et al., 2017). In fact, according to Prosser et al. (2011) and Prosser et al. (2019), most soil AOB communities are dominated by *Nitrosospira* spp., instead of *Nitrosomonas* spp. Nevertheless, unlike few specific bacterial strains (e.g., *N. europaea* and *Nitrosospira multiformis*), current knowledge of metabolism of most nitrifiers (especially archaea) is still limited, and the suitable conditions to effectively culture these microorganisms in the laboratory are unknown. Moreover, to date only *N. europaea* has been successfully transformed to emit bioluminescence for BNI assays.

### Nitrous Oxide Emissions

Low NR accessions (which effectively reduced the pace of soil nitrification) restricted N_2_O emissions by almost two times when compared with the emissions allowed by high NR accessions. Other studies have already observed that plants with high BNI ability reduce emissions of N_2_O. Subbarao et al. (2006) and Byrnes et al. (2017) reported a reduction in the cumulative flux of N_2_O by 90 and 60% after N application either in the form of synthetic fertilizer and urine, respectively.

Nitrification has been reported to be the main source of N_2_O emissions in agricultural soils in the tropics (Soares et al., 2016; Lourenço et al., 2018a, b), providing NO_3_^–^ as the substrate for heterotrophic denitrification (Kuypers et al., 2018). However, direct emissions may also occur due to incomplete oxidation of hydroxylamine under certain interactions between biotic and abiotic factors (Heil et al., 2015; Caranto et al., 2016). In fact, laboratory microcosm experiments have indicated that nitrification, instead of denitrification could be the main driver of N_2_O production in certain agricultural soils including dairy pastures (Liu et al., 2016).

In this study, the nitrification process in soil was apparently controlled by different grasses with high BNI capacity that affected the growth (and possibly activity) of AOB. According to Prosser et al. (2019), N_2_O production associated with nitrification is much higher in AOB than in AOA, and such a difference increases with NH_4_^+^ availability. Hence, to effectively control nitrification and to achieve reductions in N_2_O emissions derived from the soil it is desirable to have an impact specifically in the AOB group.

Albeit significant reductions of N_2_O emissions were observed in this study, the experimental design of the gas measurements seems insufficient to derive definitive conclusions at a larger scale due to limited temporal and spatial scales. Future studies should measure N_2_O emissions in the long-term (months) using contrasting BNI genotypes under field conditions.

### Availability of N Forms in the Soil and Uptake by Plants

According to Robertson and Groffman (2015) autotrophic nitrifiers are relatively weak competitors for N in the soil since nitrification occurs more rapidly when NH_4_^+^ availability exceeds the demand of plants and heterotrophs. Hence, BNI has been proposed as a strategy to conserve N in N-deprived ecosystems (Subbarao et al., 2015), so that plants can preserve N in the form of NH_4_^+^ while uptake from other microenvironments could occur.

No negative or positive effect was observed of NR in forage quality parameters dependent on the N nutrition status of the plant, such as shoot biomass production, CP, or N uptake. This finding may be interpreted in three ways: first, maintaining N predominantly in the form of NH_4_^+^ could represent neither an advantage nor a disadvantage from the agronomic perspective. This is contrary to what was previously proposed by Coskun et al. (2017), that the photosynthetic carbon cost of increased root exudation might decrease agricultural yield (assuming that reduced NR values are due to root-exuded BNI compounds). However, from the environmental perspective, keeping N in the form of NH_4_^+^ does represent an advantage to make the system more N conservative, since reduced availability of NO_3_^–^ may be reflected in decreased N_2_O emissions and NO_3_^–^ leaching (Subbarao et al., 2015).

A second hypothesis is that there is substantial intraspecific variation in the preference of *M. maximus* to different forms of mineral N, so that germplasm accessions that exhibited high NR may have a stronger preference to assimilate NO_3_^–^ without altering its biomass production. Previous studies explored the performance of different genotypes of *M. maximus* in nutrient solutions under different levels of NH_4_^+^: NO_3_^–^, finding that growth is optimal when both sources are present in a similar proportion (Andrade et al., 2001a, b; Santos et al., 2013). Santos et al. (2013) reported that shoot growth parameters of the cv. Aruana diminished when NH_4_^+^ exceeded 60% of the N in nutrient solution and was less affected to the excess of NO_3_^–^. Nevertheless, the root growth parameters were optimal only when both N sources were almost equally represented and dramatically declined when either NH_4_^+^ or NO_3_^–^ dominated by at least 60% of the total N in the nutrient solution.

A third alternative explanation to the poor correlation between BNI and N-related parameters is that *M. maximus* is capable of exploiting other N sources for its nutrition, such as N derived from biological nitrogen fixation (BNF). Miranda and Boddey (1987) explored the contribution of N from associated BNF in eleven ecotypes of this grass. Their results suggested that between 24 and 38% of total N accumulated in shoot tissue was derived from associated BNF, representing 5–10 kg N ⋅ ha^–1^ in 30 days.

The abovementioned mechanisms should be evaluated in future studies to provide a more comprehensive description of the interactions between genotypes of *M. maximus* and N in the soil. Several studies have proven that within this species plenty of adaptations have evolved to exploit different N sources and reduce competition for N.

### Cluster Analysis

The cluster analysis allowed the discrimination of an initial collection of 119 germplasm accessions into four subgroups with different characteristics. This analysis presents a profile of the phenotypic variability (displayed under greenhouse conditions) of the collection of *M. maximus* from which 90 candidate parental males were selected for test crosses under the new breeding program on *M. maximus* of CIAT.

No cluster with all the ideal agronomic traits was identified, but each of them may provide initial genotypes to support breeding efforts. The accessions from cluster 3 provide high productivity, high N uptake and low NR, whereas those from cluster 1 provide higher nutritional quality. In contrast, cluster 4 which comprised almost half of the collection had agronomic traits of low value according to the parameters evaluated.

Among the key germplasm accessions grouped within cluster 3 were cv. Tobiata and accession CIAT 688, both with the lowest NR in the soil, but also the commercial cvs. Mombaza and Sabanera. As mentioned before, further studies should be conducted using the cv. Tobiata to assess the mechanisms driving its high BNI ability. Its genetics could be exploited to enhance persistence and tolerance to biotic and abiotic stresses to develop an elite grass with a combination of desirable traits. Likewise, the accession CIAT 688 appears as an interesting genotype and its performance in the field should be carefully evaluated in the future.

## Final Considerations and Perspectives

In recent years, considering the increase of environmental pollution due to huge inputs of reactive N to support intensive agricultural systems, pastures of the genus *Urochloa* with high BNI capacity have been demonstrated to limit N losses due to reduced emissions of N_2_O and NO_3_^–^ leaching (Karwat et al., 2017). Nevertheless, the impact of BNI in improving N uptake and use efficiency is still under study, and to provide a more comprehensive description of this phenomenon, different N sources for plant uptake should be considered. In addition, the incorporation of BNI as a target trait in breeding schemes to release high BNI forage grasses still requires the development of a high throughput phenotyping method to assess the trait in large populations. Equally important is the elucidation of the genetic elements controlling the BNI potential in grasses.

In the light of the high BNI capacity found in certain germplasm accessions of *M. maximus*, which is a species with superior agronomic traits for sustainable intensification of cattle production, our results suggest that forage quality and provision of environmental services are not necessarily mutually exclusive. Our hypothesis that the exploration of a genetically diverse collection of the species would result in a heterogeneous distribution of the BNI trait was confirmed, and strong BNI capacity was observed which is even comparable to or exceeding the level observed with the previously tested *U. humidicola* germplasm accession (CIAT16888). At a larger scale, livestock systems based on grazing pastures with such traits could benefit by making livestock production in the tropics more N conservative while maintaining (or even increasing) animal-source protein.

## Data Availability Statement

All datasets presented in this study are included in the article/Supplementary Material.

## Author Contributions

DV carried out the experimental work, statistical analyses, and wrote the manuscript. JN coordinated the establishment of the trial. AA, JN, and JM contributed with the execution of experiments. JA, JN, and AA designed the study. JA, AA, IR, JD, and GS supervised the experimental work. All authors contributed to the analysis and interpretation of the data.

## Conflict of Interest

The authors declare that the research was conducted in the absence of any commercial or financial relationships that could be construed as a potential conflict of interest.
