# Supplementary material for: Biological Nitrification Inhibition (BNI): Phenotyping of a Core Germplasm Collection of the Tropical Forage Grass Megathyrsus maximus Under Greenhouse Conditions
Source: Front Plant Sci. 2020 Jun 12;11:820. doi: 10.3389/fpls.2020.00820 (PMC7304326; doi:10.3389/fpls.2020.00820)
Supplement: Supplementary file 1 [file Table_1.docx]

Supplementary Material

**Supplementary table 1.** BNI potential of root tissue extracts of two sets of accessions of *M. maximus* with contrasting nitrification rates. ATU: Allilthyourea units**.**

| Nitrification rates | Accession number | Specific BNI  (ATU · g of dry root^-1^) | Total BNI (ATU) |
| --- | --- | --- | --- |
| Low | cv. Tobiata 6299 | 136 | 6085 |
| Low | 688 | 141 | 3359 |
| Low | 6890 | 114 | 4252 |
| Low | 685 | 133 | 4905 |
| Low | 16025 | 126 | 4811 |
| Low | 6843 | 144 | 7098 |
| Low | 6872 | 124 | 5764 |
| Low | 16028 | 121 | 2879 |
| Low | 6461 | 138 | 5069 |
| Low | 6536 | 124 | 6464 |
| High | 16019 | 119 | 4874 |
| High | 16046 | 113 | 4586 |
| High | 16018 | 131 | 6216 |
| High | 6967 | 133 | 3563 |
| High | 6857 | 125 | 2010 |
| High | 16059 | 116 | 5023 |
| High | 26906 | 134 | 3025 |
| High | 16023 | 140 | 5078 |
| High | 6962 | 118 | 5821 |
| High | 26944 | 121 | 2943 |
| Control | *U. humidicola* | 124 | 3794 |
