# Supplementary material for: Biological Nitrification Inhibition (BNI): Phenotyping of a Core Germplasm Collection of the Tropical Forage Grass Megathyrsus maximus Under Greenhouse Conditions
Source: Front Plant Sci. 2020 Jun 12;11:820. doi: 10.3389/fpls.2020.00820 (PMC7304326; doi:10.3389/fpls.2020.00820)
Supplement: Supplementary file 2 [file Table_2.docx]

Supplementary Material

**Supplementary table 2.** Phenotypic data of 119 germplasm accessions of *M. maximus* for different agronomic and environmental traits. NR: nitrification rates, CP: crude protein, IVDMD: *in vitro* dry matter digestibility, ADF: acid detergent fiber, NDF: neutral detergent fiber.

| Accession number | Cluster | Shoot biomass (g · pot^-1^) | NR  (mg NO_3_^-^-N · kg of soil^-1^ · day^-1^) | N Uptake (mg N · pot^-1^) | CP  (%) | IVDMD (%) | ADF (%) | NDF (%) |
| --- | --- | --- | --- | --- | --- | --- | --- | --- |
| 6901 | 1 | 17.6 | 4.7 | 164.0 | 5.8 | 66.3 | 26.0 | 65.2 |
| 685 | 1 | 12.7 | 5.4 | 131.7 | 6.5 | 67.3 | 28.4 | 58.7 |
| 6954 | 1 | 17.0 | 5.6 | 168.9 | 6.2 | 63.6 | 31.6 | 62.3 |
| 6923 | 1 | 19.1 | 5.6 | 177.6 | 5.8 | 67.5 | 30.2 | 63.4 |
| 6787 | 1 | 14.6 | 5.6 | 135.0 | 5.8 | 64.6 | 30.0 | 60.6 |
| 6864 | 1 | 15.2 | 5.7 | 137.9 | 5.7 | 65.8 | 29.2 | 61.9 |
| 6900 | 1 | 10.1 | 5.8 | 81.9 | 5.1 | 64.7 | 31.1 | 61.5 |
| 6501 | 1 | 14.8 | 5.8 | 122.9 | 5.2 | 68.3 | 32.6 | 60.7 |
| 6497 | 1 | 17.0 | 5.8 | 145.2 | 5.3 | 68.0 | 30.2 | 63.6 |
| 6945 | 1 | 6.0 | 5.8 | 62.8 | 6.5 | 64.3 | 30.6 | 65.1 |
| 6175 | 1 | 18.7 | 5.8 | 151.0 | 5.1 | 65.4 | 31.6 | 60.2 |
| 6839 | 1 | 18.1 | 6.0 | 151.3 | 5.2 | 67.3 | 30.3 | 61.7 |
| 6095 | 1 | 11.6 | 6.1 | 112.3 | 6.1 | 68.1 | 30.2 | 59.5 |
| 16044 | 1 | 17.5 | 6.1 | 158.2 | 5.6 | 66.8 | 29.2 | 62.9 |
| 673 | 1 | 12.9 | 6.2 | 123.8 | 6.0 | 64.8 | 31.6 | 60.8 |
| 6983 | 1 | 12.2 | 6.2 | 113.6 | 5.8 | 65.3 | 32.5 | 62.3 |
| 16038 | 1 | 18.6 | 6.2 | 233.0 | 7.8 | 68.0 | 28.2 | 61.1 |
| 6837 | 1 | 16.2 | 6.2 | 160.2 | 6.2 | 65.0 | 29.4 | 61.0 |
| 16011 | 1 | 19.9 | 6.3 | 160.3 | 5.0 | 67.7 | 29.0 | 63.7 |
| 6094 | 1 | 18.3 | 6.4 | 160.9 | 5.5 | 66.1 | 31.3 | 61.3 |
| 16049 | 1 | 20.2 | 6.4 | 186.4 | 5.8 | 66.3 | 28.7 | 62.0 |
| 6928 | 1 | 15.3 | 6.4 | 148.9 | 6.1 | 64.7 | 32.5 | 61.7 |
| 6171 | 1 | 13.8 | 6.4 | 134.6 | 6.1 | 65.0 | 31.4 | 60.8 |
| 6906 | 1 | 18.2 | 6.4 | 195.7 | 6.7 | 64.7 | 31.1 | 62.0 |
| 6868 | 1 | 20.5 | 6.5 | 153.9 | 4.7 | 66.6 | 27.8 | 61.4 |
| 6897 | 1 | 13.3 | 6.5 | 131.8 | 6.2 | 66.7 | 28.9 | 63.3 |
| 16051 | 1 | 15.9 | 6.5 | 143.5 | 5.7 | 65.3 | 31.6 | 61.7 |
| 16003 | 1 | 17.7 | 6.5 | 182.0 | 6.4 | 67.0 | 30.3 | 63.4 |
| 622 | 1 | 16.3 | 6.5 | 139.8 | 5.4 | 66.7 | 30.6 | 60.0 |
| 6658 | 1 | 13.6 | 6.6 | 143.0 | 6.6 | 65.2 | 30.6 | 61.1 |
| 16062 | 1 | 18.8 | 6.7 | 160.8 | 5.3 | 66.6 | 31.7 | 62.4 |
| 6984 | 1 | 12.5 | 6.7 | 122.7 | 6.1 | 67.0 | 31.4 | 61.9 |
| 16060 | 1 | 14.6 | 6.7 | 127.3 | 5.5 | 67.4 | 31.4 | 61.9 |
| 26924 | 1 | 15.0 | 6.8 | 132.1 | 5.5 | 66.5 | 29.9 | 59.7 |
| 6796 | 1 | 14.6 | 6.8 | 131.3 | 5.6 | 64.0 | 30.5 | 63.4 |
| 6990 | 1 | 7.5 | 6.8 | 84.3 | 7.0 | 59.8 | 29.4 | 60.7 |
| 6915 | 1 | 15.5 | 7.1 | 135.1 | 5.5 | 64.9 | 31.7 | 61.4 |
| 6857 | 1 | 16.0 | 7.3 | 174.5 | 6.8 | 67.4 | 31.3 | 62.5 |
| 6968 | 1 | 16.4 | 7.6 | 160.1 | 6.1 | 66.1 | 28.6 | 61.3 |
| 26906 | 1 | 16.1 | 7.7 | 173.2 | 6.7 | 69.4 | 26.3 | 58.3 |
| 26925 | 1 | 16.1 | 7.9 | 135.9 | 5.3 | 65.2 | 30.3 | 61.7 |
| 26911 | 1 | 7.3 | 8.0 | 81.6 | 7.0 | 63.9 | 30.0 | 65.1 |
| 16065 | 2 | 20.0 | 8.9 | 177.2 | 5.5 | 64.5 | 32.2 | 62.5 |
| cv. Vencedor  26900 | 2 | 18.9 | 9.3 | 155.4 | 5.1 | 62.9 | 27.8 | 61.9 |
| 26360 | 2 | 20.5 | 9.4 | 202.7 | 6.2 | 65.1 | 32.0 | 62.8 |
| 26917 | 2 | 21.8 | 9.4 | 222.3 | 6.4 | 58.9 | 33.6 | 64.7 |
| 26923 | 2 | 17.1 | 9.9 | 173.1 | 6.3 | 66.6 | 32.9 | 60.9 |
| 26947 | 2 | 16.4 | 10.7 | 153.9 | 5.9 | 62.0 | 31.0 | 66.1 |
| cv. Tobiata  6299 | 3 | 24.6 | 4.6 | 186.7 | 4.7 | 65.3 | 32.9 | 63.9 |
| 688 | 3 | 22.2 | 5.0 | 179.3 | 5.0 | 67.9 | 33.0 | 63.6 |
| *U. humidicola*  16888 | 3 | 30.8 | 5.2 | 309.9 | 6.3 | 67.9 | 32.6 | 64.3 |
| 6843 | 3 | 23.1 | 5.5 | 196.5 | 5.3 | 64.0 | 33.9 | 64.5 |
| 6969 | 3 | 27.0 | 5.6 | 205.5 | 4.8 | 63.1 | 31.8 | 65.0 |
| 6500 | 3 | 23.1 | 5.6 | 235.4 | 6.4 | 67.0 | 29.1 | 61.9 |
| 16028 | 3 | 21.5 | 5.6 | 173.7 | 5.0 | 67.3 | 32.6 | 66.2 |
| 6461 | 3 | 27.9 | 5.6 | 204.0 | 4.6 | 64.1 | 33.9 | 64.5 |
| 6960 | 3 | 21.4 | 5.8 | 198.3 | 5.8 | 65.9 | 32.7 | 64.0 |
| 6831 | 3 | 20.9 | 6.1 | 184.3 | 5.5 | 64.8 | 31.5 | 63.8 |
| cv. A. Sabanera  6799 | 3 | 20.2 | 6.2 | 186.2 | 5.8 | 64.0 | 29.9 | 63.7 |
| 16034 | 3 | 28.1 | 6.3 | 190.2 | 4.2 | 64.4 | 31.4 | 65.8 |
| 16055 | 3 | 18.6 | 6.4 | 204.1 | 6.9 | 64.2 | 31.6 | 63.8 |
| 16041 | 3 | 19.5 | 6.5 | 181.5 | 5.8 | 63.8 | 33.5 | 64.9 |
| 6525 | 3 | 22.8 | 6.6 | 200.2 | 5.5 | 65.2 | 32.7 | 63.4 |
| 6963 | 3 | 24.5 | 7.2 | 183.6 | 4.7 | 65.1 | 32.5 | 62.5 |
| 16061 | 3 | 21.8 | 7.5 | 182.0 | 5.2 | 66.5 | 32.8 | 61.8 |
| 16023 | 3 | 23.9 | 7.7 | 186.7 | 4.9 | 67.5 | 31.0 | 61.6 |
| cv. Mombaza  6962 | 3 | 25.4 | 7.7 | 216.7 | 5.3 | 67.9 | 31.0 | 62.8 |
| 6890 | 4 | 17.4 | 5.3 | 113.6 | 4.1 | 64.4 | 35.0 | 65.2 |
| 6903 | 4 | 18.8 | 5.4 | 118.2 | 3.9 | 67.6 | 31.2 | 62.8 |
| 16025 | 4 | 18.9 | 5.5 | 136.9 | 4.5 | 65.3 | 31.0 | 62.2 |
| 691 | 4 | 16.0 | 5.6 | 125.4 | 4.9 | 65.9 | 32.6 | 64.3 |
| 6872 | 4 | 19.9 | 5.6 | 146.2 | 4.6 | 61.5 | 35.8 | 65.3 |
| 6842 | 4 | 15.7 | 5.6 | 113.6 | 4.5 | 65.8 | 32.9 | 64.0 |
| 6783 | 4 | 17.1 | 5.7 | 156.7 | 5.7 | 65.1 | 31.7 | 64.7 |
| 6536 | 4 | 16.1 | 5.8 | 126.9 | 4.9 | 62.8 | 34.0 | 65.0 |
| 6805 | 4 | 16.5 | 5.8 | 123.2 | 4.7 | 60.6 | 37.8 | 65.6 |
| 6929 | 4 | 17.0 | 5.8 | 133.6 | 4.9 | 63.9 | 32.0 | 65.1 |
| 6840 | 4 | 18.4 | 5.8 | 134.1 | 4.6 | 65.7 | 31.4 | 63.7 |
| 6898 | 4 | 19.4 | 5.8 | 150.4 | 4.8 | 63.7 | 33.1 | 64.7 |
| 6836 | 4 | 14.7 | 5.9 | 128.4 | 5.5 | 62.9 | 34.4 | 61.7 |
| 6996 | 4 | 16.5 | 6.0 | 133.5 | 5.1 | 66.8 | 33.4 | 62.4 |
| 6893 | 4 | 18.1 | 6.0 | 134.7 | 4.7 | 64.7 | 32.8 | 64.6 |
| 16005 | 4 | 16.9 | 6.0 | 129.5 | 4.8 | 63.6 | 32.8 | 65.7 |
| 6948 | 4 | 19.3 | 6.1 | 155.3 | 5.0 | 64.6 | 30.4 | 65.0 |
| 16048 | 4 | 18.4 | 6.1 | 145.3 | 4.9 | 64.9 | 31.5 | 62.6 |
| 6784 | 4 | 16.3 | 6.2 | 135.7 | 5.2 | 65.7 | 32.1 | 62.9 |
| 16069 | 4 | 19.6 | 6.3 | 149.1 | 4.8 | 65.2 | 33.8 | 63.7 |
| 6912 | 4 | 18.4 | 6.4 | 162.6 | 5.5 | 65.1 | 31.7 | 64.1 |
| 6866 | 4 | 15.7 | 6.4 | 120.1 | 4.8 | 63.9 | 34.6 | 62.5 |
| 16064 | 4 | 16.4 | 6.4 | 147.5 | 5.6 | 64.5 | 33.8 | 63.3 |
| 36000 | 4 | 14.9 | 6.4 | 119.6 | 5.0 | 64.6 | 31.4 | 62.8 |
| 16035 | 4 | 20.2 | 6.5 | 153.1 | 4.7 | 65.0 | 32.2 | 61.2 |
| 6826 | 4 | 20.1 | 6.6 | 147.3 | 4.6 | 65.6 | 31.7 | 63.7 |
| 6927 | 4 | 19.1 | 6.6 | 122.9 | 4.0 | 64.1 | 33.7 | 63.3 |
| 6944 | 4 | 15.6 | 6.6 | 142.9 | 5.7 | 64.6 | 32.8 | 62.8 |
| 16068 | 4 | 14.9 | 6.7 | 119.8 | 5.0 | 65.1 | 30.7 | 62.6 |
| 16027 | 4 | 16.0 | 6.7 | 117.3 | 4.6 | 64.9 | 32.3 | 64.3 |
| 16058 | 4 | 14.3 | 6.7 | 127.6 | 5.6 | 64.4 | 31.3 | 64.5 |
| 16004 | 4 | 17.2 | 6.7 | 131.7 | 4.8 | 63.1 | 33.1 | 65.5 |
| 6918 | 4 | 18.2 | 6.7 | 137.4 | 4.7 | 64.4 | 31.5 | 63.8 |
| 6986 | 4 | 18.9 | 6.7 | 160.6 | 5.3 | 67.0 | 32.8 | 62.5 |
| 16017 | 4 | 17.7 | 6.8 | 162.6 | 5.7 | 67.3 | 32.2 | 64.3 |
| 16054 | 4 | 15.4 | 6.8 | 150.6 | 6.1 | 65.1 | 33.0 | 64.5 |
| 6855 | 4 | 18.2 | 6.9 | 142.2 | 4.9 | 65.9 | 31.7 | 63.2 |
| 16071 | 4 | 16.0 | 6.9 | 128.5 | 5.0 | 65.3 | 31.7 | 64.0 |
| cv. Massai  16021 | 4 | 18.1 | 6.9 | 144.9 | 5.0 | 63.4 | 33.8 | 66.8 |
| 16036 | 4 | 16.7 | 6.9 | 160.9 | 6.0 | 62.8 | 34.3 | 63.8 |
| 26937 | 4 | 15.5 | 6.9 | 115.9 | 4.7 | 66.5 | 32.2 | 63.6 |
| 16019 | 4 | 16.0 | 7.0 | 159.3 | 6.2 | 68.4 | 36.9 | 64.7 |
| 16046 | 4 | 16.2 | 7.0 | 115.7 | 4.5 | 63.7 | 33.3 | 62.5 |
| 16057 | 4 | 14.7 | 7.0 | 127.7 | 5.4 | 65.2 | 31.7 | 64.6 |
| 6955 | 4 | 20.1 | 7.1 | 164.7 | 5.1 | 65.2 | 32.8 | 63.3 |
| 16018 | 4 | 16.3 | 7.1 | 108.8 | 4.2 | 64.2 | 32.0 | 64.2 |
| 26942 | 4 | 14.4 | 7.2 | 126.3 | 5.5 | 63.4 | 33.2 | 65.2 |
| 6967 | 4 | 18.4 | 7.2 | 159.3 | 5.4 | 67.3 | 32.3 | 64.5 |
| 16059 | 4 | 10.9 | 7.5 | 102.7 | 5.9 | 63.6 | 33.8 | 62.0 |
| 6981 | 4 | 13.6 | 7.9 | 110.5 | 5.1 | 65.5 | 34.3 | 63.0 |
| 26944 | 4 | 15.4 | 8.1 | 108.7 | 4.4 | 62.2 | 34.2 | 63.9 |
| 6982 | 4 | 20.3 | 8.7 | 142.6 | 4.4 | 66.0 | 33.0 | 63.7 |
